# Assessing the impact of COVID-19 on tuberculosis detection and treatment in healthcare facilities across Addis Ababa, Ethiopia: A comprehensive mixed-method, multi-center study

**DOI:** 10.1371/journal.pone.0311408

**Published:** 2024-10-03

**Authors:** Beshir Bedru Nasir, Oumer Sada Muhammed, Melaku Tileku Tamiru, Legese Chelkeba

**Affiliations:** Department of Pharmacology and Clinical Pharmacy, School of Pharmacy, College of Health Sciences, Addis Ababa University, Addis Ababa, Ethiopia; Norbert Wiener University, PERU

## Abstract

**Background:**

Ethiopia faces a significant burden of Tuberculosis (TB), being one of the high-burden countries, and the emergence of the Coronavirus Disease 2019 (COVID-19) has become a dominant health concern, particularly in resource-limited settings. The repercussions of COVID-19 on TB care are evident, leading to a surge in undiagnosed TB cases, challenges in medication adherence, and an escalation of drug resistance. Consequently, a thorough assessment of the impact of COVID-19 on TB care becomes imperative to devise a tailored program for managing TB amidst future pandemics, natural disasters, and conflict crises.

**Methods:**

A mixed-methods study design was utilized, encompassing a randomly selected 10 health centers (HCs) and 3 hospitals among government owned 98 HCs and 5 hospitals in Addis Ababa, Ethiopia. All TB patients who were on follow-up during the study period were included. The study period was from March 4, 2020, to December 4, 2020, with the corresponding period of March 4, 2019, to December 4, 2019, serving as the baseline for comparison. Quantitative data were gathered from TB patients’ medical registries, laboratory registries, and treatment follow-up charts. Complementary qualitative data were acquired through in-depth interviews. Both qualitative and quantitative data were collected from January 17, 2022 to May 13, 2022.

**Results:**

Following the onset of the pandemic, there was a notable and statistically significant decline in both the detection of TB cases and the number of positive results across all study sites. Bacteriological TB tests reduced from 5837 to 2126 patients, and TB-positive cases decreased from 500 to 218, representing declines of 63.6% and 56.4%, respectively. The overall number of TB patients undergoing treatment also experienced a decrease from 1431 to 1051, marking a 26.6% reduction. Additionally, there was a 10% increase in the proportion of extra-pulmonary TB cases. The impact of the pandemic extended to TB treatment outcomes, with adverse effects on cure rates, death rates, loss of follow-up, and medication adherence. The apprehension of contracting COVID-19 and the implementation of isolation measures contributed to a decline in healthcare-seeking behaviors among patients, fostering negative perceptions and practices among healthcare workers. The challenges further exacerbated due to a shortage of personal protective equipment, a lack of rapid diagnostic test tools, clinical presentations resembling COVID-19, and a shift in government policies. These factors collectively posed significant obstacles to effective TB care during the pandemic.

**Conclusion:**

The profound impact of COVID-19 on critical TB care service indicators, including TB detection, treatment initiation, and treatment outcomes, underscores the need for immediate and collaborative measures. It is imperative to implement strategies that ensure the resumption of all TB care services concurrently with efforts to control COVID-19. A comprehensive and coordinated approach is essential to mitigate the adverse effects of the pandemic on TB management and safeguard public health.

## Introduction

Coronavirus Disease 2019 (COVID-19) has emerged as a worldwide health and economic threat, leading to a substantial increase in cumulative incidence globally. Anticipating the severity of the projections, hospitals worldwide have been proactively establishing additional critical care surge capacity and, in the process, limiting routine patient access to care for other conditions such as tuberculosis (TB) [1, 2]. Ethiopia is identified as one of the 30 high-burden countries for TB, encompassing drug-resistant TB [3]. According to the World Health Organization (WHO) 2016 report, there was an elevated incidence; with an annual estimated TB incidence of 177/100,000 population and a death rate of 25 per 100,000 population in the same year. Consequently, Ethiopia recorded notifications for 125,836 new TB cases and 702 drug-resistant TB cases in 2016 [4].

Ethiopia shoulders a significant share of the global burden of infectious diseases, and until July 2020, the pattern of COVID-19 in the country was in the early stages of the epidemiological curve. The Ethiopian government has undertaken substantial efforts to control the spread of the disease [1]. Despite limited testing capacity, as of September 28, 2022, there have been 493,534 confirmed COVID-19 cases and 7,572 deaths, based on only 5.3 million tests conducted. A multicenter study encompassing low and middle-income countries (LMIC) uncovered significant challenges faced by patients and healthcare providers in accessing healthcare facilities in many LMIC [5]. In China, a study revealed that the COVID-19 epidemic primarily impacted TB notification and follow-up examinations, potentially leading to a surge in demand for TB services in the near future [6]. Similarly, research conducted in Italy indicated that COVID-19 resulted in decreased TB detection, elevated default rates, and increased mortality among TB patients [7]. In Africa, studies reported that presumptive pulmonary TB and detected TB cases were declined by 31.2% and 28.0% in Kenya [8] and 35% and 34% in Nigeria respectively [9].

The global dominance of the COVID-19 pandemic has eclipsed all other health concerns. The significance of its impact on TB, a persistent leading cause of death from a single infectious disease worldwide, cannot be overstated. The repercussions encompass a surge in undiagnosed TB cases in LMICs, placing strain on TB control programs due to resource diversion and an inevitable shift in health system priorities. These challenges are anticipated to result in a decline in the quality of TB care and worsened outcomes. The similarity in clinical presentation and diagnostic overlap could exacerbate the stigmatization of TB patients, particularly in LMICs, fueled by the fear of COVID-19 [10]. The Ethiopian government undertook significant efforts to mitigate the impact of the disease, implementing measures such as restricting public gatherings, closing schools, and sealing borders during the pandemic crisis. Certain health facilities that traditionally provided TB care and treatment services were repurposed as COVID-19 isolation and treatment centers. This redirection of human and material resources from TB to COVID-19 initiatives had repercussions on TB case finding and care [1]. Given the country’s lack of comprehensive data, there is a limited understanding of the impact of COVID-19 on TB patient diagnosis, treatment start, and clinical outcomes. Hence, the primary objective of this study is to thoroughly examine the detection rate and clinical outcomes of TB patients during both the pre-COVID-19 and intra-COVID-19 periods within public health centers (HCs) and hospitals in Addis Ababa, Ethiopia.

## Methods

### Study settings

The investigation was carried out in a randomly selected 10 HCs and 3 hospitals among 98 HCs and 5 hospitals situated in Addis Ababa, Ethiopia. The specific study sites encompassed Kolfe Keranio (Wored-3) HC, Teklehaymanot HC, Addis Raey HC, Lideta HC, Dilfire HC, Afinchober HC, Jagama Kello Memorial HC, Chefe HC, Kuasmeda (Woreda 09) HC, Hiwot Amba HC, Zewditu Memorial General Hospital, Minilik General Hospital, and Yekatit-12 Teaching Hospital. All these healthcare facilities are government-owned, providing services to patients from both Addis Ababa and the surrounding Oromia regions. Each HC has the capacity to cater to approximately 40,000 people, while each hospital is anticipated to serve a population ranging from 1 to 1.5 million individuals. The selection of these HCs and hospitals was based on the availability of anti-TB medications, which are provided by the government as part of a programmed drug distribution, free of charge. Consequently, all selected healthcare facilities are equipped to offer TB treatment services. Patients eligible for TB treatment can conveniently refill their medication from the nearby HC, as anti-TB medications are readily available in all HCs and the designated hospitals. HCs, being the preferred locations for anti-TB medication refills, are considered primary points of access. In situations where patients face complications requiring further referral, other hospitals and private health settings in the country can redirect patients to a nearby HC or hospital for follow-up and medication refill.

### Study design and period

This research employed both quantitative and qualitative study designs, with a focus on a comparative cross-sectional study design for the quantitative aspect. The qualitative section was a simple descriptive explanatory study to support the quantitative findings.

The primary aim was to assess the impact of COVID-19 on various aspects of TB management, including detection, treatment initiation, treatment outcomes, medication adherence, and follow-up. The study period spanned from March 4, 2020, to December 4, 2020. To establish a baseline for comparison, a corresponding period during the previous year, from March 4, 2019, to December 4, 2019, was considered. These seasons were chosen to evaluate changes during the COVID-19 pandemic, taking into account the various restrictions implemented to control the spread of the virus. Both qualitative and quantitative data were collected from January 17, 2022, to May 13, 2022.

### Sampling and sample size determination

In Addis Ababa, ten out of the 98 HCs and three out of the five government-owned general hospitals were randomly selected for inclusion in the study. The sample comprised all patients undergoing TB treatment at the selected HCs and hospitals. For the qualitative aspect, two healthcare professionals from each facility, specifically those working in cough outpatient clinics, were chosen for in-depth interviews. However, due to saturation of in-depth interviews, reaching a total of 21 healthcare workers out of the initially planned 26, these individuals were included in the qualitative study.

### Study variables

In the quantitative section, the study focused on several variables: the number of patients undergoing bacteriological tests for TB (GeneXpert/ Acid-Fast Bacillus), the number of confirmed TB cases, the total number of patients initiated on anti-TB medication, medication adherence for Directly Observed Therapy (DOT), and TB treatment outcomes.

### Data collection instrument and collection process

Quantitative data were obtained from TB patients’ medical registries, laboratory registries, and treatment follow-up charts for DOT therapy. In-depth interviews were employed to assess healthcare workers’ perceptions and practices regarding TB detection and treatment initiation. All interviews were recorded, and notes were taken during sessions lasting 30–45 minutes. Five clinical pharmacy graduates were enlisted for quantitative data collection, while two pharmacists were recruited for the qualitative component. Training by the principal investigator preceded data collection, and a pre-test in one health center ensured the appropriateness and completeness of the data collection tool, with necessary modifications implemented accordingly.

### Data analysis

Quantitative data were analyzed using Statistical Package for the Social Sciences (SPSS) version 26 software. Descriptive statistics, such as frequencies and proportions, were employed to summarize study characteristics. Paired t-tests were utilized to assess the significance of mean differences in selected variables, comparing changes from the baseline. Qualitative data underwent manual analysis using themes and participant verbatim quotes. The thematic analysis followed a deductive approach, starting with predetermined codes for the data set. Additionally, inductive coding was applied to capture issues not covered by the deductive analysis or not fitting the predetermined themes, generating new themes by identifying patterns in the data set.

### Ethical considerations

Ethical clearance and approval for the study were obtained from the Addis Ababa University, College of Health Sciences, School of Pharmacy Ethical Review Board (Ref No. ERB/SOP/387/142021), Addis Ababa City Administration Health Bureau and Ethical Review Board (A/A/12092/227), and Yekatit-12 Teaching Hospital (Protocol no. 076/22) before commencing data collection. Approval letters were submitted to the study facilities to ensure compliance with ethical standards and written informed consent was obtained from the study participants for qualitative section. For retrospective data extraction from medical records, confidentiality was ensured by avoiding all personal identifiers during data collection and the data was reported in aggregate. Moreover, the consent for retrospective data collection was waived by the three ethics committee and approval letters were received before data collection.

## Results

### Quantitative study

This study included all TB patients on follow-up in the 10 HCs and 3 general hospitals.

The number of TB detections through bacteriological examination (GeneXpert test) and the positive results for the TB test demonstrated a decline following the onset of the COVID-19 outbreak. This impact was evident across nearly all study sites. Prior to the pandemic, a total of 5837 patients underwent testing, resulting in 500 confirmed TB-positive cases, with subsequent initiation of anti-TB medications. Conversely, during the pandemic within the same duration, only 2126 patients were tested, reflecting a 63.6% reduction and 218 patients were confirmed and treated, indicating a 56.4% reduction (see Table 1 for details).

**Table 1:** Pre and post COVID-19 GeneXpert tests and results to detect TB cases.

Pre COVID-19 GeneXpert lab test and results				Post COVID-19 GeneXpert lab test and results		
	Total tests	Positive results	Negative results	Total tests	Positive results	Negative results
Site 1	432	42	390	189	33	156
Site 2	194	21	173	39	4	35
Site 3	180	20	160	105	17	88
Site 4	271	10	261	80	4	76
Site 5	232	15	217	92	2	90
Site 6	116	6	110	38	9	29
Site 7	325	30	295	135	28	107
Site 8	70	2	68	13	1	12
Site 9	99	10	89	15	0	15
Site 10	345	14	331	100	4	96
Site 11	1102	108	994	639	56	583
Site 12	1321	87	1234	162	16	146
Site 13	1150	135	1015	519	44	475
**Total**	**5837**	**500**	**5337**	**2126**	**218**	**1908**

Site 1: Kolfe keranio HC, Site 2: Teklehaiymanot HC, Site 3: Addis Raey HC, Site 4: Lideta HC, Site 5: Dilfire HC, Site 6: Afinchober HC, Site 7: Jagama kello memorial HC, Site 8: Chefe HC, Site 9: Kuasmeda (Woreda 09) HC, Site 10: Hiwot Amba HC, Site 11: Zewditu Memorial general hospital, Site 12: Yekatit-12 teaching hospital, Site 13: Minilik general hospital

Concerning the total TB patients on follow-up, which encompasses individuals diagnosed within the study setting and those referred from other private health facilities or government hospitals showed a significant decline. Thus, at the baseline, a total of 1431 TB patients were on follow-up, whereas after the advent of COVID-19, only 1051 patients remained on TB follow-up, reflecting a 26.6% decline across the study sites (refer to Table 2 for details). Furthermore, the proportion of pulmonary TB cases accounted for 707 (49.4%) during the pre-COVID period, and this figure decreased to approximately 415 (39.5%) during the pandemic period, as illustrated in Table 2.

**Table 2:** Number and types of total TB patient on follow-up during pre and post COVID-19.

	Follow-up status of TB patients (Pre-COVID-19)			Follow-up status of TB patients (Post-COVID-19)		
Study site	*Total TB patients*	*Pulmonary TB*	*Extra pulmonary TB*	*Total TB patients*	*Pulmonary TB*	*Extra pulmonary TB*
Site 1	107	56	51	85	49	36
Site 2	89	60	29	50	32	18
Site 3	114	74	40	55	46	9
Site 4	27	15	12	39	22	17
Site 5	65	41	24	46	25	21
Site 6	34	19	15	42	17	25
Site 7	63	28	35	42	15	27
Site 8	18	8	10	13	4	9
Site 9	23	12	11	19	8	11
Site 10	35	18	17	34	9	25
Site 11	463	226	237	303	145	158
Site 12	13	9	4	12	4	8
Site 13	380	141	239	311	39	272
**Total**	**1431**	**707**	**724**	**1051**	**415**	**636**

Site 1: Kolfe keranio HC, Site 2: Teklehaiymanot HC, Site 3: Addis Raey HC, Site 4: Lideta HC, Site 5: Dilfire HC, Site 6: Afinchober HC, Site 7: Jagama kello memorial HC, Site 8: Chefe HC, Site 9: Kuasmeda (Woreda 09) HC, Site 10: Hiwot Amba HC, Site 11: Zewditu Memorial general hospital, Site 12: Yekatit-12 teaching hospital, Site 13: Minilik general hospital

HC: Health center

### TB treatment outcomes among the selected HCs and hospitals

**Fig 1:**
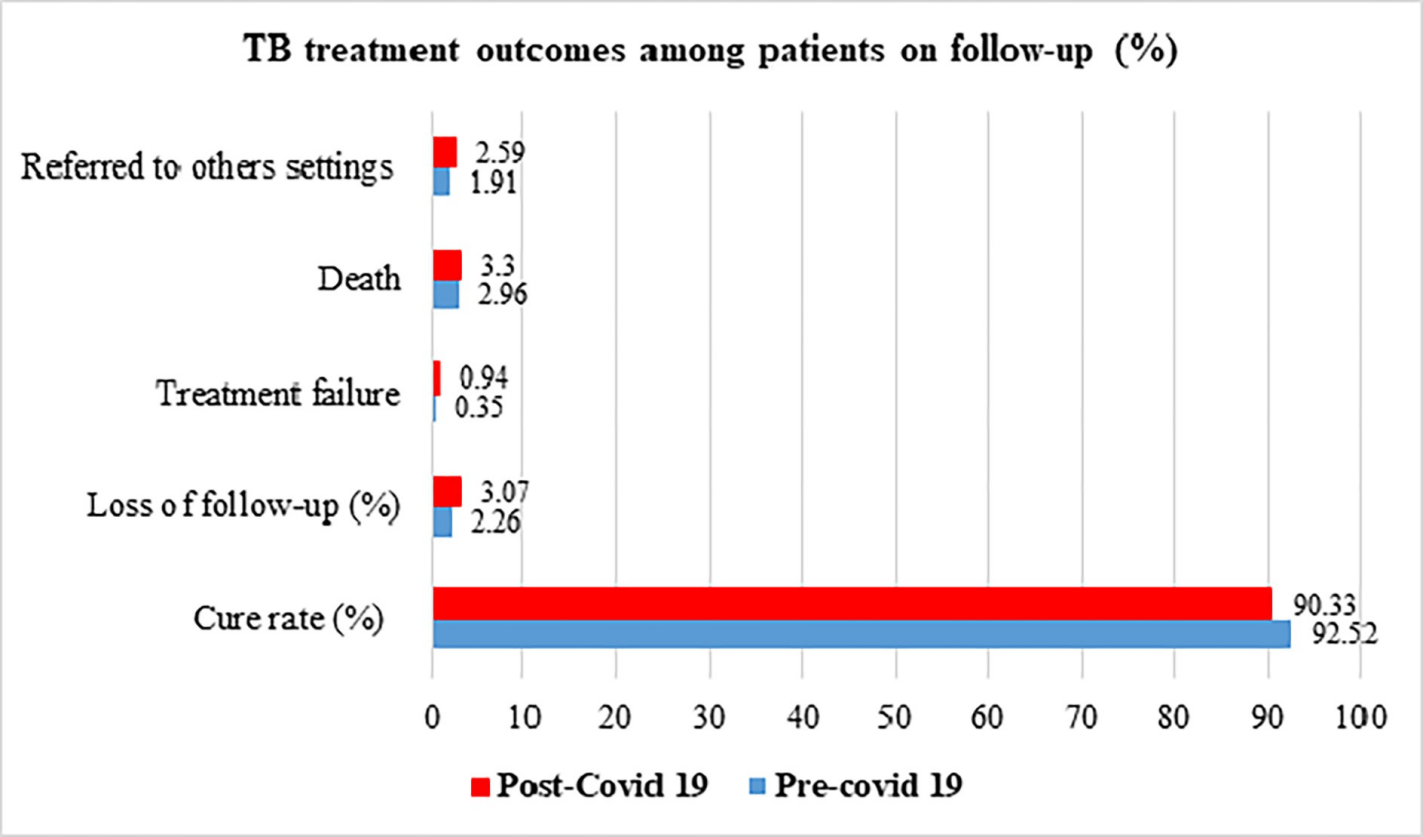
Comparison of pre and post COVID-19 TB treatment outcomes.

The treatment outcomes of TB patients exhibited varied findings among the selected HCs, as detailed in Table 3. Notably, the treatment outcomes before the pandemic slightly surpassed those observed during the pandemic. Specifically, the cure rate stood at 92.52% before the pandemic, marginally higher than the 90.33% recorded during the pandemic. Similarly, the death rate was 2.96% in the pre-COVID period, compared to 3.3% during the COVID-19 period (see Fig 1 for graphical representation). It is important to note that this finding excludes the study hospitals, as a substantial number of patients from these hospitals were transferred to other primary facilities for anti-TB medication refills. Consequently, treatment outcomes and medication adherence were not assessed for the hospitals in this context.

**Table 3:** Treatment outcomes of TB patients on follow-up during pre and post COVID-19.

Treatment outcomes of Pre-COVID-19							Treatment outcomes of post COVID-19					
Study site	Total patients with TB on follow-up	Cured	Loss of follow-up	Treatment failure	Death	Referred to others	Total patients with TB on follow-up	Cured	Loss of follow-up	Treatment failure	Death	referred to others
Site 1	107	97	3	1	3	3	85	81	0	0	1	3
Site 2	89	81	5	0	2	1	50	43	3	2	2	0
Site 3	114	111	1	0	1	1	55	50	2	0	3	0
Site 4	27	27	0	0	0	0	38	37	1	1	0	1
Site 5	65	62	0	1	1	1	46	42	1	0	2	0
Site 6	34	34	0	0	0	0	42	39	1	1	1	0
Site 7	63	55	1	0	3	4	42	34	1	0	1	6
Site 8	18	15	1	0	1	1	13	10	2	0	0	1
Site 9	23	19	0	0	4	0	19	17	0	0	2	0
Site 10	35	31	2	0	2	0	34	30	2	0	2	0
Total	**575**	**532**	**13**	**2**	**17**	**11**	**424**	**383**	**13**	**4**	**14**	**11**

Site 1: Kolfe keranio HC, Site 2: Teklehaiymanot HC, Site 3: Addis Raey HC, Site 4: Lideta HC, Site 5: Dilfire HC, Site 6: Afinchober HC, Site 7: Jagama kello memorial HC, Site 8: Chefe HC, Site 9: Kuasmeda (Woreda 09) HC, Site 10: Hiwot Amba HC

HC: Health center

### Anti-TB medications adherence during pre and post COVID-19 among the selected HCs

Out of the 575 patients under follow-up during the baseline, a noteworthy 96.9% demonstrated good adherence to anti-TB medications. However, during the pandemic, among 424 patients, the percentage with good adherence slightly decreased to 92.7%, indicating a decline of 4.2%.

### Trends in TB detection and follow-up among patients with TB during pre- and post-COVID-19 pandemic

**Fig 2:**
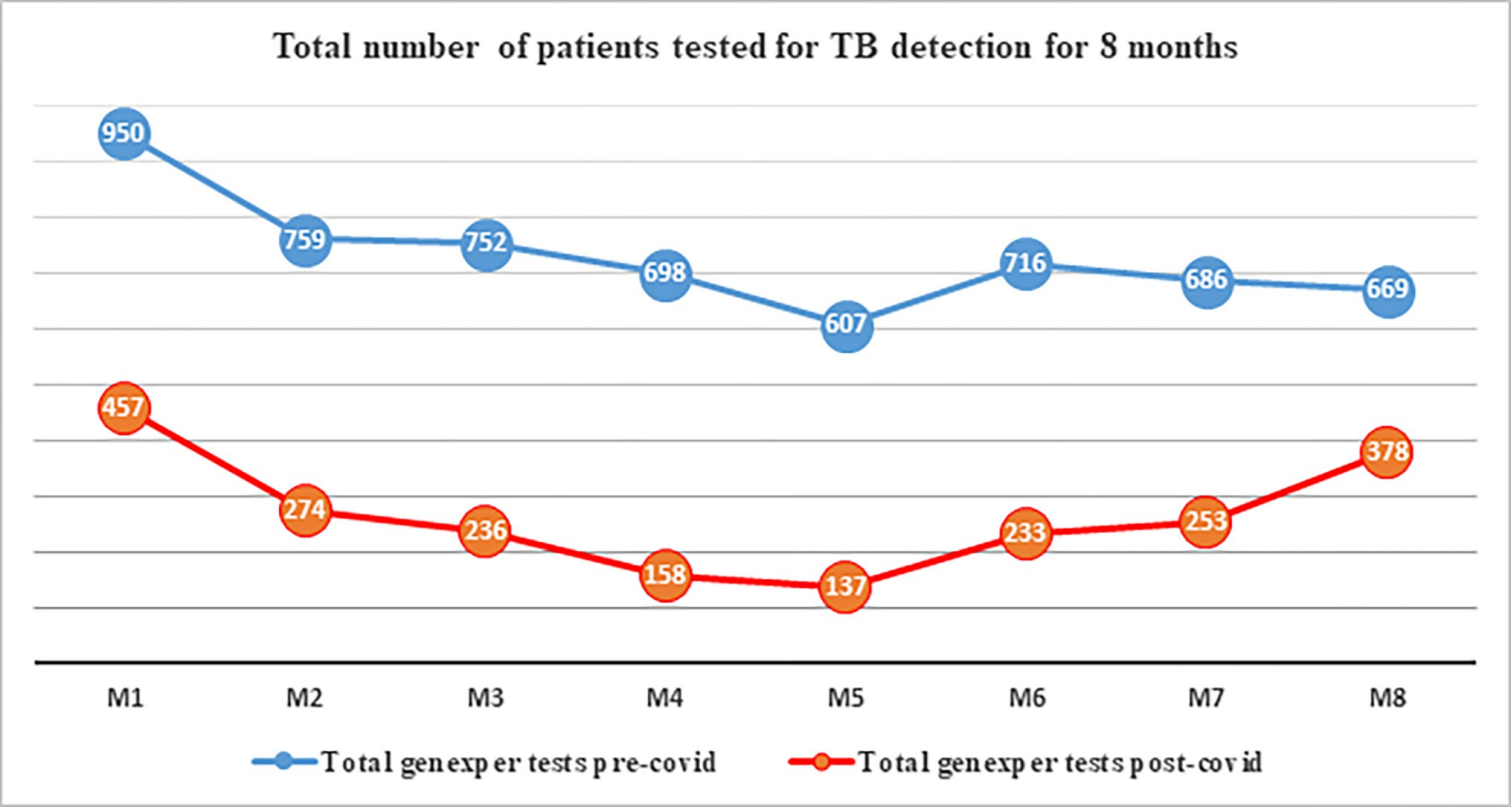
Pre and post COVID-19 Genexpert tests for TB detection trends over 8 months. M1: March 13^rd^ to April 13^rd^_,_ M2: April 14^th^ to May 13^rd^, M3: May 14^th^ to June 13^rd^, M4: June 14^th^ to July 13^rd^, M5: July 14^th^ to August 13^rd^, M6: August 14^th^ to September 13^rd^, M7: September 14^th^ to October 13^rd^, M8: October 14^th^ to November 13^rd^. (The 8 months were 2019 for pre COVID-19 and 2020 for post COVID-19).

**Fig 3:**
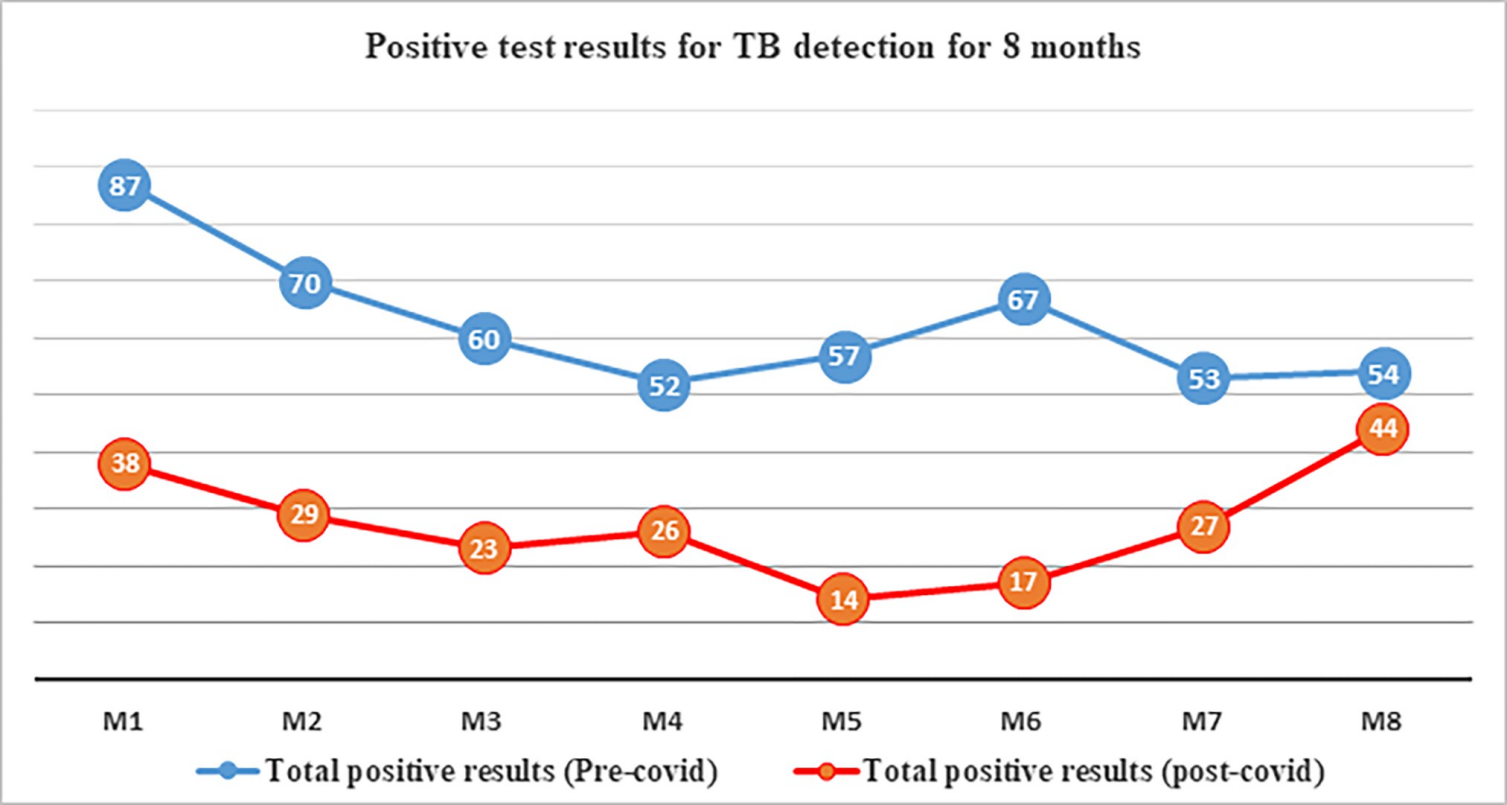
Pre and post COVID-19 positive test results of TB detection trends over 8 months. M1: March 13^rd^ to April 13^rd^_,_ M2: April 14^th^ to May 13^rd^, M3: May 14^th^ to June 13^rd^, M4: June 14^th^ to July 13^rd^, M5: July 14^th^ to August 13^rd^, M6: August 14^th^ to September 13^rd^, M7: September 14^th^ to October 13^rd^, M8: October 14^th^ to November 13^rd^. (The 8 months were 2019 for pre COVID-19 and 2020 for post COVID-19).

**Fig 4:**
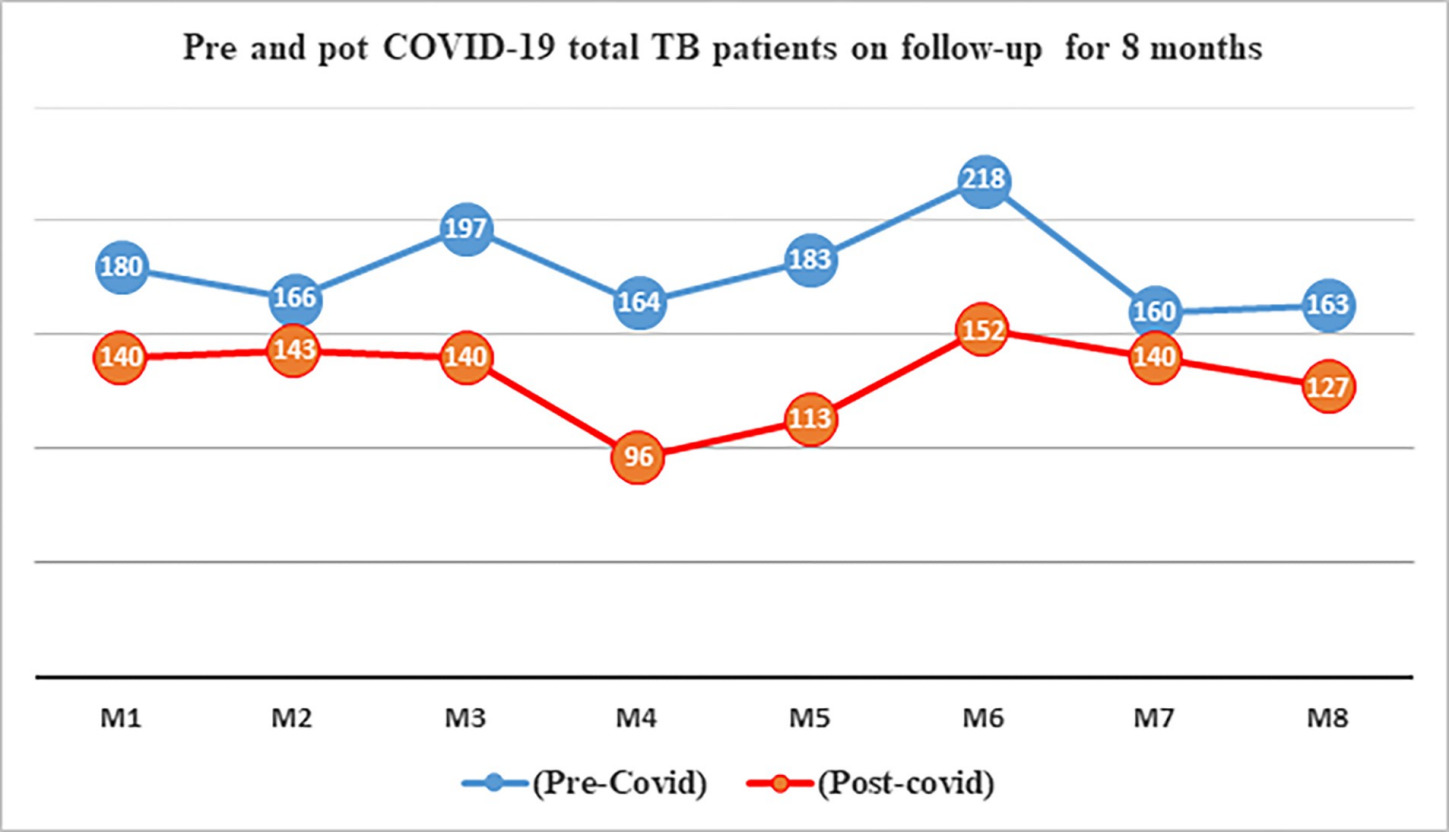
Pre and pot COVID-19 total TB patients on follow-up trends over 8 months. M1: March 13^rd^ to April 13^rd^_,_ M2: April 14^th^ to May 13^rd^, M3: May 14^th^ to June 13^rd^, M4: June 14^th^ to July 13^rd^, M5: July 14^th^ to August 13^rd^, M6: August 14^th^ to September 13^rd^, M7: September 14^th^ to October 13^rd^, M8: October 14^th^ to November 13^rd^. (The 8 months were 2019 for pre COVID-19 and 2020 for post COVID-19).

The examination of the number of patients tested for TB across corresponding months during the study periods revealed substantial numerical disparities, as illustrated in Fig 2. Likewise, both the number of positive results and the total count of patients on TB follow-up were notably lower during the COVID-19 pandemic in comparison to the baseline, as depicted in Figs 3 and 4, respectively.

### Comparison of pre and post COVID-19 differences for selected variables

In the statistical comparison of pre and post-COVID-19 TB care, all variables exhibited a statistical significance with p < 0.05. The mean differences for total TB testing, and total TB patients on follow-up were 284.46, 21.69, and 29.23, respectively (refer to Table 4 for detailed results).

**Table 4:** A T-test mean comparison of pre and post COVID-19 differences for selected variables.

	Descriptions	Paired differences			
		Mean differences	95% CI		P value
			Lower	Upper	
Pair 1	Total tests before the pandemic	284.46	98.09	472.83	**0.006**
	Total tests during the pandemic				
Pair 2	Total positive results before the pandemic	21.69	3.64	39.74	**0.022**
	Total positive results during the pandemic				
Pair 3	Total TB patients on follow-up before the pandemic	29.23	1.21	57.25	**0.042**
	Total TB patients on follow-up during the pandemic				

### Qualitative study

#### Socio-demographic characteristics of key informants

A total of 21 key informants (KIs) participated in the interviews, with ages ranging from 25 to 56 years. The distribution of key informants included four nurses, ten health officers, and seven medical doctors (Table 5).

**Table 5:** Socio-demographic characteristics of key informants (n = 21).

Description	Category	N (%)
Gender	Male	9(42.9)
	Female	12 (57.1)
Age (Years)	25–30	8(38.1)
	31–35	8(38.1)
	36–40	1(4.8)
	>40	4(19.0)
Profession	Nurse	4(19.0)
	Health officer	10(47.6)
	Physician	7(33.3)
Academic qualification	Bachelor’s degree	16(76.2)
	Master’s Degree	2(9.5)
	Medical Specialty	3(14.3)
Work experience (Years)	<5	5(23.8)
	5–10	10(47.6)
	>10	6(28.6)

### Attitudes and practices of health care providers towards TB care during COVID-19 pandemic

A manual thematic analysis was followed for the analysis and interpretation of results under three core thematic categories through both deductive and inductive approaches. The three major themes were: Attitude and practice of the participants towards TB care during the pandemic; Challenges of TB care during COVID-19 pandemic and the current status of TB care during COVID-19 pandemic. The three themes are described below.

#### Theme 1: Attitudes and practices of the participants towards TB care during the pandemic

The prevailing sentiment among the majority of participants was a significant fear of COVID-19 infection. This apprehension was primarily rooted in concerns about the potential consequences of contracting COVID-19, such as the risk of death, transmission to family members, and the prospect of isolation. Participants commonly perceived COVID-19 as a highly lethal and easily transmissible disease, leading many to acknowledge that this fear impacted their ability to deliver optimal care for TB cases. According to the participants, this negative influence extended beyond TB care, affecting various health services due to shifts in the healthcare setting. The disruption in healthcare services was further exacerbated by the reassignment of many healthcare workers to COVID-19 centers, while others were compelled to stay at home. This collective impact contributed to an overall disturbance in the healthcare service delivery system. Furthermore, patients, aiming to avoid COVID-19 testing and subsequent isolation, resorted to concealing respiratory symptoms, a behavior that potentially led to a decline in the detection rate of TB cases.

*Honestly, in the early stages of the pandemic, there was a profound fear of potential death from COVID-19. This fear influenced us not to inquire about TB symptoms or conduct TB screenings; instead, the priority was to isolate the patient and take measures to protect ourselves. [Health Officer with 8 years of experience]*

*During that period*, *it was exceptionally challenging*. *If a patient presented with a cough*, *fear would set in*, *and my immediate instinct was to leave the room*, *hindering further communication and potentially resulting in missed TB cases*. *Whenever encountering a patient with a cough*, *the immediate assumption was more aligned with COVID-19 rather than TB*. *Consequently*, *individuals exhibiting cough symptoms were strongly encouraged to leave the Outpatient Department (OPD) promptly due to the prevailing fear of COVID-19*. *[Health Officer with 28 years of experience]*

Participants emphasized that the information circulated in the media during the pandemic was alarming, contributing to heightened anxiety among health workers in relation to COVID-19. The perception was that the information might have been exaggerated, possibly stemming from the absence of established scientific evidence on the severity of the infection and its optimal management at that early stage.

Prior to the onset of the pandemic crisis, TB patients followed a treatment model based on DOT for duration of 8 weeks. However, during the pandemic, there was a notable shift in the treatment approach. Medications were dispensed on a weekly, bi-weekly, tri-weekly, or quad-weekly basis, deviating from the traditional DOT model. This alteration raised concerns about potential treatment failure, the emergence of resistance, and an increased risk of loss of follow-up among TB patients.

*The shift in the DOT approach to a monthly schedule has effects on patients’ adherence. There is uncertainty about whether they consistently took their medicines. Strikingly, post-COVID-19, there has been a notable increase in the number of TB relapses. Unlike the pre-pandemic scenario where relapses were anticipated after 2–3 years, they are now occurring immediately after treatment completion. Even patients who initially showed sputum negativity at the fifth month of therapy are developing TB lymphadenitis. This phenomenon is attributed to poor adherence stemming from the absence of Directly Observed Therapy (DOT). [Experienced Nurse with 13 years in the field]*

On the contrary, a minority of participants perceived COVID-19 as an opportunity to control TB transmission. They viewed the interventions implemented to control COVID-19 as crucial measures to curb the transmission of TB. Some participants mentioned that they did not experience any fear and believed that it would not adversely affect TB care during the pandemic.

*Despite the shortage of Personal Protective Equipment (PPE) and prevailing fear, I made a conscious effort not to overlook any potential TB cases. However, it’s important to emphasize that this task cannot be accomplished by a single individual alone. I genuinely swear that I am dedicated to this work for my own well-being, and I have never allowed fear to alter my established practices. Nonetheless, I acknowledge that meaningful changes require collective efforts beyond my individual contribution. [Health Officer with 10 years of experience]*

Amid the pandemic, a significant proportion of participants did not contemplate TB as a potential cause for respiratory symptoms. Any presentation of respiratory symptoms immediately led to the assumption that COVID-19 was the primary culprit. Consequently, essential investigative steps such as taking a comprehensive medical history, conducting a physical examination, and other diagnostic measures were neglected. In this context, TB received little attention, and TB OPD clinics were converted to serve only suspected COVID-19 patients.

Furthermore, there was a broader systemic issue where the government, the Ministry of Health, and stakeholders predominantly directed their focus towards COVID-19. Unfortunately, this singular emphasis resulted in the oversight of other critical services, including those related to TB.

*It was indeed a challenging period, with the government, the Ministry of Health, and various stakeholders, including non-governmental organizations (NGOs), placing a significant emphasis on prioritizing COVID-19. This prioritization resulted in the neglect of TB across various health services. [Physician with 5 years of experience]*

However, few numbers of respondents did consider TB as a potential cause for respiratory symptoms. Recognizing subtle differences in the clinical presentation of the two diseases, TB was duly considered, leading to the initiation of TB tests and subsequent treatment. These respondents also highlighted that specific training sessions were conducted for a select group of health professionals on TB care during the COVID-19 pandemic. As healthcare workers who commenced their careers with a solemn oath, they expressed a commitment unaffected by fear, ensuring that their clinical practices remained steadfast.

*TB was typically detected if a patient exhibited symptoms such as weight loss, poor appetite, and a persistent cough lasting for at least 2 weeks. In contrast, COVID-19 manifested as symptoms of acute illness, including fever, loss of taste, sore throat, and joint pain. These distinct symptom profiles, including variations in the type of cough, allowed healthcare professionals to differentiate between TB and COVID-19. [Health Officer with 13 years of experience]*

Having worked in a TB clinic for numerous years, my concern for TB persisted. I could discern differences in symptoms between TB and COVID-19. COVID-19 symptoms were notably acute, often accompanied by shortness of breath. In contrast, TB presented with chronic symptoms such as night sweats. [Nurse with 13 years of experience]

*During the initial phase*, *patients were hesitant to articulate their illness*, *primarily driven by fear*. *Despite this*, *our routine practice*, *which included a dedicated cough clinic*, *continued*. *Comprehensive screenings such as ESR*, *Chest X-ray*, *and GeneXpert were routinely conducted*, *acknowledging the importance of assessing not only COVID-19 but also other potential respiratory conditions*. *[Physician with 2 years of experience]*

#### Theme 2: Challenges of TB care during COVID-19 pandemic

Patients refrained from visiting HCs out of fear of contracting COVID-19, fearing isolation and other associated risks. Individuals experiencing respiratory symptoms avoided seeking healthcare due to concerns about potential isolation and quarantine if suspected or diagnosed with COVID-19. This avoidance significantly contributed to a decrease in TB screening and detection rates. Additionally, healthcare providers’ own fears of COVID-19 infection acted as a barrier to effective TB case detection and care. Throughout the pandemic, the government’s primary focus was on managing COVID-19, resulting in most healthcare facilities being repurposed for COVID-19 care. This reconfiguration, coupled with a shortage of PPE for healthcare providers, created a challenging environment for maintaining adequate protection during patient interactions. Logistic issues further complicated the ability to provide essential health commodities in healthcare settings, exacerbating the challenges faced during this period.

*We encountered numerous challenges during the pandemic. Many patients chose to stay at home, even when experiencing severe respiratory symptoms, driven by the fear of a potential COVID-19 diagnosis and subsequent isolation. Even when they sought medical attention, patients were often reticent about explaining their respiratory symptoms. Other notable challenges included a lack of government focus on TB amid the pandemic and the insufficient availability of Personal Protective Equipment (PEP), hindering the ability to conduct comprehensive physical examinations. [Physician with 8 years of experience]*

In the past, there was an established TB screening program in the community, with support from NGOs. However, during the pandemic, all these activities were abruptly halted, and the focus shifted entirely to addressing COVID-19. The challenges faced for TB care included a notable lack of awareness and information among both the community and healthcare providers. This shift in priorities and diminished awareness added complexity to the effective management of TB during the pandemic.

*Several community-based programs for TB detection and care, which had prior support from the government and NGOs, faced a significant setback during the outbreak. The focus of all efforts shifted entirely to the control of COVID-19. Consequently, community-based TB campaigns and screenings were abruptly interrupted, resulting in a substantial impact on the provision of TB care. [Health Officer with 8 years of experience]*

A notable challenge was the scarcity of COVID-19 tests to rule it out and explore other potential etiologies. The symptoms closely resembled those of COVID-19, complicating the differentiation. Additionally, patients faced difficulties in providing any TB history, including information about previous TB infections and potential contact with TB patients. This lack of comprehensive medical history further complicated the diagnostic process.

*In the initial stages, the absence of a rapid COVID-19 test posed a challenge in confirming the lack of COVID-19 infection and exploring alternative etiologies. [Physician with 6 years of experience]*

#### Theme 3: Current status of TB care with COVID-19 pandemic

Presently, there has been significant improvement in TB case detection and care. However, notable gaps persist in community screening and the resumption of previous TB care programs. The increased awareness among both patients and healthcare workers is contributing to a rise in the identification of TB cases, with enhanced laboratory screening to pinpoint TB patients. The practices of healthcare workers in optimizing TB detection and treatment are on an upward trajectory, with a gradual resumption of crucial procedures such as thorough physical examinations and comprehensive history-taking.

*Despite the initial decrease in emphasis on TB care due to the emergence of COVID-19, the current scenario has reverted to a state similar to the pre-COVID-19 period. [Health Officer with 6 years of experience]*

*Currently*, *patient follow-up in this health center has returned to a more normal routine*, *although the predominant focus remains on COVID-19*. *Fortunately*, *the prevailing fear has diminished*, *and healthcare providers are actively conducting physical examinations to identify chest findings*. *[Nurse with 8 years of experience]*

Therefore, there is an ongoing need for concerted efforts in Ethiopia, given its high burden of TB. While the global focus has shifted towards COVID-19, it is crucial to emphasize that TB demands sustained and continuous attention, support, follow-up, and ongoing initiatives, including training and the development of comprehensive guidelines.

*Presently, there is an observed improvement in patients’ attitudes, and the number of TB screenings has increased. However, the predominant focus on respiratory diseases remains on COVID-19. To alter this trend and optimize TB care, we anticipate the need for training, orientations, and ongoing follow-up. [Health Officer with 5 years of experience]*

## Discussion

The COVID-19 pandemic has caused devastating effects across global health, with TB services bearing a disproportionate impact [1]. This study aims to elucidate the ramifications of COVID-19 on TB care during the pandemic. The findings reveal a significant decline in overall TB detection, treatment initiation, and treatment success.

In this investigation, the total presumptive TB cases (based on bacteriological TB tests) experienced a notable decrease of 63.3%, while bacteriologically confirmed TB cases saw a reduction of 56.4%. This reduction in TB detection surpassed rates observed in studies conducted in Kenya (31.2%) [8], Nigeria (35%) [9], and Sierra Leone (12.7%) [11]. Possible contributors to this variance include differences in study duration, the emphasis on TB care during the pandemic, the healthcare service capacity, and the magnitude of TB and COVID-19 burden during the study period.

The initiation of anti-TB medication witnessed a decrease of 26.6% during the pandemic, with an additional shift seen in the proportion of pulmonary TB, marking a 10% reduction. Notably, the impact on extra-pulmonary TB case finding, particularly in hospitals, remained relatively less affected. This aligns with a WHO report [12] indicating a 21% decrease in TB notification across countries with the highest TB burden. However, our study reports a milder reduction in confirmed TB notification rates compared to studies in Uganda (43%) [13], Nigeria (34%) [9], and South Africa (33%) [14]. The lower rate of TB detection reduction in our study could be attributed to disparities in study duration and data collection methods. While previous studies relied on aggregate secondary data reports, our approach involved direct data collection from individual health facilities. Furthermore, variations in the timing of data collection during the pandemic, with some studies focusing on the early stages [9, 14] and others exploring the impact of specific public policies like lockdowns on TB notification [13], may account for the differences in reported reductions.

The reduction in confirmed TB cases may stem from various factors, encompassing a decline in TB case detection or testing during the pandemic. This decline could be attributed to perspectives held by patients, healthcare workers, and government health policies. Insights into these factors were elucidated in the qualitative findings, revealing a decrease in patients’ health-seeking behavior owing to the fear of COVID-19 infection within healthcare facilities and apprehensions about isolation or quarantine. Healthcare workers expressed concerns regarding patients presenting with respiratory symptoms, perceiving all such symptoms as potential indicators of COVID-19 and thereby diverting attention away from TB. Furthermore, the government’s focal shift towards the pandemic resulted in the reorganization of hospitals and HCs for COVID-19 care and isolation, the re-allocation of healthcare professionals to COVID-19 care, and movement restrictions that impeded visits to health facilities. Additionally NGOs that played a crucial role in supporting TB care, including awareness creation and community-based TB detection, redirected their efforts to pandemic control.

It may seem reasonable to attribute the decline in TB incidence during the pandemic to factors such as reduced population mobility, social distancing, and mask-wearing. However, this assumption may not necessarily hold true, as the significant decrease in patient flow to health facilities and TB tests suggests under diagnosis rather than a true decline in incidence. The observed drop in TB notification rates could be indicative of missed TB diagnoses during the pandemic, leading to a substantial buildup of undetected TB cases, fostering ongoing TB transmission and increasing rates of latent TB infection and associated mortality. According to a WHO report, the impact of reductions in TB detection and care in 2020 is estimated to result in half a million excess TB deaths, setting the global TB mortality rate back by a decade to 2010 levels [12]. In light of these challenges, leveraging virtual care, digital health technologies, and integrating COVID-19 and TB screening may emerge as the most effective strategies to mitigate the pandemic’s impact on TB services.

The trend observed throughout the study period indicates a decreasing impact on bacteriologically confirmed TB cases and the total number of patients on follow-up. This trend serves as a positive indicator that TB care has been progressively improving, and there is a likelihood that routine TB care will resume concurrently with the management of COVID-19. This optimistic outlook is further supported by insights gained from the qualitative exploration, where a subset of key informants highlighted a diminishing impact of COVID-19 over time. Therefore, according to the qualitative data gathered, there has been a positive trend in practices related to TB case detection and laboratory screening. This improvement is attributed to increased awareness among patients and healthcare workers. The practices of healthcare workers in optimizing TB detection and treatment are on an upward trajectory, as reflected in the resumption of physical examinations, thorough history taking, and other essential procedures for TB care. However, challenges persist, particularly in community screening, reinstating previous TB care programs, and receiving support from non-governmental organizations (NGOs) for sustaining continuous and comprehensive TB care services.

The treatment outcomes of TB experienced a slight decline during the COVID-19 pandemic, with the cure rate dropping from 92.52% to 90.33%, the death rate increasing from 2.96% to 3.3%, and the loss of follow-up rising from 2.26% to 3.07%. Despite this decline, the observed treatment outcomes were comparatively better than those reported in studies conducted in Korea (from 89.4% to 84.5%) [15] and Sierra Leone (from 55.6% to 46.7%) [11]. The improved outcomes in the present study could be attributed to differences in the study population. The evaluation of treatment outcomes focused exclusively on TB patients in primary healthcare and OPD settings, with less severe TB cases, and only drug-susceptible cases were included, potentially contributing to a higher cure rate.

Despite the existence of disagreement on the role of DOT in TB control, it has shown to reduce the acquisition and transmission of drug resistant TB [16]. DOT also shown to improve treatment outcomes especially in low and middle income countries with low health literacy [17, 18]. Thus, in adherence to the Ethiopian national TB treatment guideline, DOT should be administered on a daily basis for the first 2 months. However, due to the pandemic, most HCs altered the DOT approach to refilling medication every week, every 2 weeks, or on a monthly basis depending on the patients’ condition. This modification could potentially impact medication adherence and elevate the risk of treatment failure. In the present study, medication adherence through DOT in the studied HCs was initially high at 96.9%, but during the COVID-19 period, it declined to 92.7%. It’s essential to note that the decrease in medication adherence might be underestimated, given the altered DOT therapy and the challenge of ensuring that patients take the medication at home. Poor medication adherence is linked to an increased risk of treatment failure and resistance. According to key informants, there was an observed rise in the number of TB relapses after COVID-19, occurring immediately after treatment completion.

### Strengths and limitations of the study

The study employed both quantitative and qualitative research methods, providing a comprehensive understanding of the impact of COVID-19 on TB care. This dual approach enhances the validity and depth of the findings. Furthermore, the inclusion of various healthcare professionals, including nurses, health officers, and medical doctors, as key informants contributes to a holistic perspective on the challenges and practices related to TB care. The study also has some limitations: The study was conducted in Addis Ababa, Ethiopia. The findings may not fully represent the diversity of TB care challenges across different regions of the country, limiting the generalizability of the results. In addition, the study focuses on healthcare providers’ perspectives, and the patient experience during the pandemic is not extensively explored. Including patient feedback could enhance the study’s comprehensiveness. Despite these limitations, the study provides valuable insights into the challenges and adaptations in TB care during the COVID-19 pandemic, offering a foundation for future research and targeted interventions.

## Conclusion

This study underscores the substantial adverse impact of COVID-19 on various facets of TB care services, including TB detection, treatment initiation, treatment outcomes, and medication adherence. Key factors contributing to compromised TB care during the pandemic encompassed patients’ apprehension of COVID-19 infection and isolation, healthcare workers’ altered perceptions and practices, and governmental policy changes. This situation may have led to an accumulation of undetected TB cases, fostering ongoing transmission, increased rates of latent TB infection, drug resistance, and associated mortality [19].

## Supporting information

S1 File Raw data. (XLSX)

S2 File Raw data for hospitals. (SAV)

S1 File Raw data for HCs. (XLSX)

## Abbreviations

COVID-19: Coronavirus Disease 2019
DOT: Directly Observed Therapy
HCs: Health Centers
HO: Health Officers
LMIC: Low and Middle-Income Countries
NGOs: Non-Governmental Organizations
OPD: Outpatient Department
PEP: Personal Protective Equipment
TB: Tuberculosis
WHO: World Health Organization
